# Patients' perceptions of their doctors' notes and after‐visit summaries: A mixed methods study of patients at safety‐net clinics

**DOI:** 10.1111/hex.12641

**Published:** 2017-11-02

**Authors:** Brittaney M. Belyeu, Jared W. Klein, Lisa M. Reisch, Sue Peacock, Natalia V. Oster, Joann G. Elmore, Sara L. Jackson

**Affiliations:** ^1^ Department of Medicine Kaiser Permanente West Los Angeles Medical Center Los Angeles CA USA; ^2^ Department of Medicine University of Washington School of Medicine Seattle WA USA

**Keywords:** electronic medical records, patient portal, primary care, safety‐net clinic

## Abstract

**Background:**

Patients are increasingly offered electronic access to their doctors' notes, and many consistently receive paper After‐Visit Summaries. Specific feedback from patients about notes and summaries are lacking, particularly within safety‐net settings.

**Design:**

A mixed methods study

**Setting and Participants:**

Patients with poorly controlled diabetes attending two urban safety‐net primary care clinics in Washington State.

**Methods:**

Patients read their own most recent clinic note and After‐Visit Summary, then completed a brief survey followed by a focus group discussion (3 groups in a large general medicine teaching clinic and 1 in an HIV/AIDS clinic) about their perceptions of the clinic note and After‐Visit Summary.

**Results:**

Twenty‐seven patients participated; 70% were male, 41% were Black, 48% were unemployed or disabled, 56% reported fair/poor health, and 37% had accessed the electronic patient portal. A majority of patients felt their note content was useful (89%); a minority reported that their notes were not accurate (19%), had too much medical jargon (29%), or were too long (26%). Themes identified from the discussions included reliance on the provider to explain confusing content; a desire for more rather than less detail; and perceived inaccuracies, particularly in heavily templated notes. In each focus group, one or more portal users were enthusiastically willing to teach other patients.

**Conclusions:**

The majority of focus group participants at this safety‐net site had not accessed the electronic patient portal, but those who had were willing to promote the portal benefits and assist others. Patients identified specific opportunities to improve clinic notes and After‐Visit Summaries.

## 1 INTRODUCTION

To date, 15 million patients at more than 40 different medical centres, clinics, or health networks in the United States now have electronic access to their own notes.^1^, ^2^ Historically, doctors' written patient progress notes serve multiple purposes including the following: recording the patient's plan of care; communication between health professionals; a legal record of the encounter; and documenting care and services provided to the patient.^3^ Increasingly doctors' notes can be accessed via secure online patient portals and allow a platform for communicating with patients. Patients who read their notes report having a better understanding of their health,^1^, ^4^ and relatively few patients are concerned about privacy (37%)^1^, ^5^ or feel that reading their clinic notes causes confusion, worry or offence (1% to 8%).^1^, ^4^ Qualitative feedback from patients about their note content has been positive, but patients in safety‐net settings may have different perceptions of their doctors' notes.^6^ Also, vulnerable populations are less likely to access patient portals,^7^ which raises concerns about whether this technological intervention for engaging patients might actually increase health‐care disparities due to a “digital divide.”^8^, ^9^, ^10^ A better understanding of electronic note access among vulnerable populations, who may have relatively limited computer access or lower health literacy, is needed.

In addition to doctors' notes, After‐Visit Summaries are another avenue for communicating health information to patients. This document, summarizing actionable instructions for patients after an office visit, is postulated to improve communication and coordination of care, and can be shared with patients' family members or other health‐care providers.^11^ After‐Visit Summaries are provided to approximately 50% of patients completing outpatient visits in the United States,^12^ including via electronic access to the health record. A study of patients' perceptions of After‐Visit Summaries among predominantly White, college‐educated primary care patients identified multiple opportunities to improve the usefulness of the content.^13^ Another study among a majority Hispanic population found that patients liked receiving a clinical summary, and that the quantity of content did not affect patient recall or satisfaction.^14^ Feedback from safety‐net populations about use of electronic portals, and both doctors' note and After‐Visit Summary content will help optimize opportunities for communication and engagement.

Prior to obtaining electronic access to doctors' notes, focus groups among a diverse, vulnerable patient population in 2010 found that patients' knowledge about their own health record was low.^15^ While they responded positively to the idea of gaining access to their electronic clinic records, some worried about loss of privacy and disruption of the patient‐provider relationship.^15^ Now that these patients have had electronic access to their own doctors' notes for over 2 years, we conducted a follow‐up study using mixed methods, consisting of four focus groups and a brief survey to obtain patients' feedback about reading their own doctors' notes and After‐Visit Summaries, in addition to self‐reported use of the electronic patient portal.

## 2 METHODS

This mixed methods study obtained survey data followed by audio‐taped qualitative focus group discussions. It was conducted at Harborview Medical Center, an urban safety‐net teaching medical center affiliated with the University of Washington in Seattle WA, with patients from two teaching clinics. The clinics provide primary care to the hospital's mission population of the medically underserved, including patients who are incarcerated, homeless and under‐ or uninsured.^16^, ^17^ The Adult Medicine Clinic is a large general internal medicine teaching clinic serving diverse patients with complex chronic conditions, many with mental health or substance use disorders. The Madison Clinic is a primary care teaching clinic for patients with HIV/AIDS. Both clinics participated in the original OpenNotes study in 2010,^1^, ^18^ and both clinics' patients have been invited to access their notes since October 21, 2014 via an electronic patient portal. We administered a printed survey and conducted four focus groups within these two clinics between March and July of 2016.

### 2.1 Patient recruitment

We used purposive sampling for participant recruitment. Eligible patients had poorly controlled diabetes (haemoglobin A1C levels > 9), as identified using clinic diabetes registries. Diabetic patients were chosen because the clinics were specifically interested in patients' experiences with their diabetes care. We excluded patients who had not had an encounter with a provider in the clinic within the preceding 12 months or whose primary language was not English. Among eligible diabetes patients, clinic staff members then identified patients who were likely to attend and actively participate in a group discussion. Research staff members attempted to contact 81 patients by phone and successfully contacted 43 of these patients; 31 expressed interest in participating, and 27 attended the groups.

### 2.2 Human subjects protection

The University of Washington Institutional Review Board deemed the focus groups exempt from review as the goal was to improve clinical care delivery. The purpose of the focus groups was reviewed verbally with the participants, and each participant provided written informed consent. Transportation was reimbursed, lunch was provided, and each participant received a $25 grocery coupon.

### 2.3 Patient questionnaire and focus group discussions

Before the focus group discussions began, we asked participants to read a printed copy of their own most recent clinic note and After‐Visit Summary from their primary care provider. We then asked patients to complete a survey that included demographic questions and five‐point Likert‐scale questions evaluating their use of the electronic patient portal and their perception of their printed doctor's note and After‐Visit Summary (Appendix S1).

Three of the authors (BB, SJ, LR) facilitated the groups, which were audio‐taped and lasted approximately 2 hours. The focus group interview guide, developed by the authors, began with questions about patients' experience using or not using the electronic health portal. The facilitators then asked participants questions about their doctor's notes and the After‐Visit Summaries using a semi‐structured format with open‐ended questions about what patients liked, what could be improved, and the clarity of the content. The focus group discussions were audio recorded and transcribed.

### 2.4 Analysis

Three study authors (BB, SJ, JK) reviewed the transcribed focus groups and performed iterative rounds of analysis using the immersion‐crystallization technique^19^ to identify key themes; we used the principles of (i) “grounded theory” to generate theories from participants who have personally experienced clinic notes and After‐Visit Summaries, and (ii) “participatory action research” to break down barriers between facilitators and participants to produce suggested improvements to clinic notes and After‐Visit Summaries.^20^ Ideas were organized by theme along with representative quotations. The investigators met to resolve discrepancies in their independent reviews, build consensus around key domains and match representative quotations to appropriate themes. Only themes that were independently identified by all three reviewers are presented. Summary statistics of participant demographics and opinions were calculated using SAS 9.4 (Cary, NC).

## 3 RESULTS

Table 1 shows patient demographics and self‐reported health status for the 27 participating patients. Seventy per cent of participants were male, 41% were Black, 48% were unemployed or disabled, 37% had a high school education or less, and 56% reported fair/poor health.

**Table 1:** Demographic characteristics and self‐reported health status of patient participants

Characteristic	Number	%^a^
Total	27	100
Gender		
Female	8	30
Male	19	70
Age (yrs)		
35‐55	11	41
56‐65	7	26
66‐75	8	30
Racial/Ethnic background		
Black or African‐American	11	41
White or Caucasian	11	41
American Indian or Alaska Native	2	7
Latino	3	11
Education level		
Some high school or less	3	11
High school graduate or equivalent	7	26
Some college	10	37
College graduate or post graduate	6	22
Employment status		
Employed	3	11
Retired	10	37
Unemployed or disabled	13	48
Self‐reported health rating		
Very good or good	11	41
Fair	10	37
Poor	5	19

a Per cent may not equal 100 due to one respondent missing data in age, education, employment and self‐reported health categories.

**Table 2:** Themes of patients related to electronic patient portal use, the doctor's note and the After‐Visit Summary

Electronic patient portal
Lack of access or familiarity with technology
Concerns about lack of privacy/security
Users are strong advocates and want to help their peers
Doctor's note
Difficult to understand
Medical jargon and abbreviations
Reliance on provider to explain what patient does not understand
Preference for detail
Doctor's note and After‐Visit Summary
Contains inaccurate or outdated information that patients would like to help correct
Used as a reference document
For patient
For family/caregivers
For outside 4medical providers

### 3.1 Electronic patient portal use

**Figure 1:**
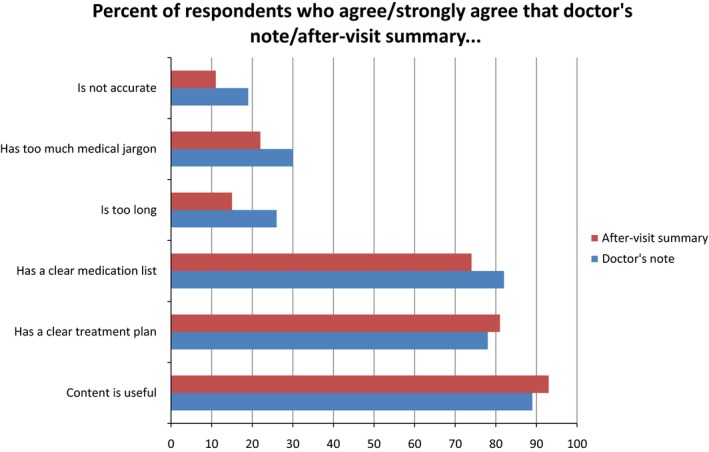
Patient (n = 27) perceptions after reading their last primary care doctor's note and After‐Visit Summary

About half of 27 focus group participants reported not previously having used the electronic patient portal (n = 14 had not accessed, n = 10 had accessed, n = 3 did not respond). The most commonly reported reason for not using the portal was lack of access to an electronic device (n = 7), while other reasons included concern about privacy (n = 4), lack of interest (n = 2), concern that the contents would be worrisome (n = 2), or too complicated (n = 1), or other (n = 3). Among the 10 portal users, the most common uses were to view laboratory results (n = 9), to view visit notes (n = 8) and to send messages to the care provider (n = 6). Less common uses were to view radiology results (n = 3) and to request pharmacy refills (n = 2). The majority of patients felt the content of both the notes and After‐Visit Summaries were useful and clear, while a minority noted opportunities for improvement (Figure 1).

### 3.2 Themes related to accessing the electronic patient portal

The themes identified from the discussions about accessing the portal included two barriers to using this tool, and a potentially effective strategy to facilitate portal use.

#### 3.2.1 Lack of access or familiarity with technology

Many participants expressed that a lack of access or familiarity with technology was the main reason they were not using the electronic patient portal. These patients expressed not having access to a computer; not knowing how to use technology; lack of interest in learning how to use computers; or preferred to obtain information in ways that they were familiar with. Some participants with barriers to computer use expressed strongly that they had no interest in it, while others were interested in learning how to access the portal and their information.

Because I don't have a computer. My budget don't allow me to have a computer and I'm old school. You never know when it goes out. So you need to know old school. A6 (letter indicates group and number indicates participant within the group)

I just don't care for it. I don't care for electronic stuff. C1

I've not done [the electronic patient portal] mostly because I'm not tech savvy, I have a computer in my house, but I haven't gotten into [the portal]… A8

#### 3.2.2 Concerns about privacy or security

Additionally, some participants were concerned that use of the electronic patient portal would potentially compromise the privacy and security of their personal health information, and some did not like the idea of communicating with staff members they did not know.

…but the hacking part is always with me. [Referring to a news story:] You probably know about the laptop that was taken? That's scary. Hopefully, nothing happens. That's the only disadvantage I see. D2

They [the messages] all go into a nursing bank, then they decide if it's going to get any further. So I quit using it, I won't use it for that…Yeah, I don't know who they are. I'm kind of that way. C3

#### 3.2.3 Experienced portal users advocate for its use

Although many focus group participants had not previously used the electronic patient portal, more experienced patient portal users were vocal advocates.

I can copy them over and put them in OneNote or Evernote or whatever if I really needed them. I'm a big fan of knowing where my stuff is. To me, that's easier than giving me this [printed After Visit Summary]. Like I said, I'll go home and have a stack of these, and the first thing I'll do is jump on [the electronic patient portal]. C3

During the focus groups, portal users offered to teach others how to access the portal and become familiar with its features. In each group, there were participants who expressed interest in learning how to use the portal. In response to a participant who did not know how to access the electronic portal, one participant asked:

Well, but if we were in a group I could show you how, couldn't I? Everybody can help everybody else here, that's the whole point. B2

### 3.3 Themes related to perceptions of the doctor's note

Three major themes surfaced when discussing the doctor's note. Patients found parts of their notes difficult to understand, often relied on providers to interpret their notes, but still preferred that doctors' notes remain detailed rather than being shortened or simplified.

#### 3.3.1 Notes are sometimes difficult to understand

After reviewing their own printed clinic note, it became clear that participants—many of whom had not previously seen a clinic note—had difficulty understanding its contents. This was primarily attributable to two primary factors: (i) the presence of medical jargon and (ii) unfamiliarity with medical abbreviations.

I didn't go to school to learn these words and I have no interest in learning these words. I want it dummied down. You tell me what ACEI is, type of thing. Don't throw that on my paperwork and then hand it to me and expect me to know it. Write it out in layman's terms if you're going to hand me notes. B4

I find it really helpful, but like one of the guys said, a lot of times I don't understand it, because [my doctor] uses all sorts of medical terms that I have no idea what she's talking about. D6

#### 3.3.2 Patients rely on providers to explain content or preferred direct verbal communication

Despite difficulty understanding the information in the clinic note, some participants did not want their notes simplified. Instead, they wanted their doctor to interpret the contents of notes.

My doctor lets me know what's wrong. I might not understand all these abbreviations, but he'll tell me, and then I just do what I do. But the information I think should stay there [in the note]. B8

I've had to ask my doctors a few times what's this, this and this, and they just kind of write it on the side. C3

Some patients expressed the desire to obtain information verbally from the doctor and had no interest in trying to understand the note content.

I told him the first day I saw him, I said, ‘you’re the doctor and I'm the patient, so that's the way this relationship will work. I'm not a doctor so you're going to have to tell me what to do.' So pretty much from what he tells me when we're in clinic is fine with me. Since I know all of this is in [the clinic note] and I don't understand three‐quarters of it, why bother? D2

#### 3.3.3 Patients prefer detailed notes

Lastly, rather than preferring that details be removed to simplify clinic notes, participants preferred to keep notes detailed and to instead communicate directly with their provider about documentation that was difficult to understand or needed clarification.

I would rather see it and disregard it on my own than somebody doing that for me. I don't want somebody to keep it simple for me. C2

Oh yeah, give me all the information. Because I'm going to go look it up otherwise. So at least here it is… This is more bulk, more data. I think it's good. C2

### 3.4 Themes related to both the clinic note and After‐Visit Summary

Two additional themes were identified regarding both the doctor's note and the printed After‐Visit Summary. Namely, patients described using both clinical documents for their own reference and shared them with others; and they also identified inaccurate or outdated information in both clinical documents.

#### 3.4.1 Patients use clinical documents for reference

Patients reported using both the doctor's note and After‐Visit Summary as a reference for themselves and to communicate with family, caregivers and other medical personnel about active medical problems, medical history and follow‐up plans.

I like my summaries because I can go back and revisit them, so I like that. B6

This is good information to have and it would be good for my kids to know and have. I don't have to explain every detail to them, they would know what is what and how to go about it and take care of it.A6

I used to carry a medication list and now I don't have to, because it's always there. So I carry this in my purse all the time. When I get a new one, I just carry the new one. Because if for any reason, if anything happens to you and you can't tell anybody anything, you have all this information right here. That's a good thing to me. A7

I kind of like the fact that they have so much information on here that—like I said, I live in an area that I have to take this with me if I have to go to an emergency room. So if I'm not well enough to go over my history with the ER doctor… B3

#### 3.4.2 Clinical documents contain inaccurate or outdated information

Some participants noted inaccuracies in their doctor's note or the After‐Visit Summary. The errors tended to occur in auto‐populated or templated sections, such as the medication list and the patient problem list.

Probably 90 percent of what's been prescribed to me, all listed on here, I don't take and haven't been taking for years. C2

Some of them are now obsolete, so what I have done is the last time I went to see the last doctor and they say are you still taking this, this, and this, and I'll say no, take that off and take that off. So I kind of have to cull through it myself at the time so that it doesn't appear anymore. That's something that I have to do. D4

I was reading this [the problem list] and I saw something on here that's no longer the case, so it shouldn't be on there. C4

## 4 DISCUSSION

Over one‐third of the focus group participants attending these safety‐net clinics had accessed the electronic health portal. While many expressed difficulties with access to computer portals and reservations about computer use, others were vocal advocates for the portal. Enthusiastic users volunteered to assist others with electronic access and espoused the benefits of being able to see and use their own information. Another key study finding was that participants reported the content of their recent doctor's note and After‐Visit Summary was useful (89 and 93%, respectively) on the survey. However, during group discussion, common themes included identification of inaccuracies and confusing medical jargon. This suggests that the more nuanced group dialogue highlighted specific opportunities for improvement of notes and After‐Visit Summaries not captured by the survey. Additionally, a subset of discussants reported relying on their physicians to interpret and explain the written documentation. Given this mixed feedback about the usefulness of notes and After‐Visit Summaries, providing patients access to their records online is not an end in itself, but rather a starting point to improve the accuracy and clarity of clinical documentation and a mechanism to better engage patients in their continuing care.

There is concern that open access to electronic health information may augment existing health disparities by widening the “digital divide” between those with and without Internet access.^21^ Poor health outcomes are more likely when patients are elderly or have lower socioeconomic status, education levels and health literacy; these attributes are also associated with lack of Internet access.^22^ In addition, a recent US study found that patients who are Black people, Hispanic, or who have a lower education level are less likely to report being offered electronic access to their health information by their health‐care provider.^23^ Thus, the patients who stand to benefit the most from access to their health information have multiple barriers to surmount in obtaining it. Many of our focus group participants described lack of online access and/or apprehension about technology use, and many had not accessed the electronic portal. Given these barriers, further work is required not only to provide instruction for vulnerable populations in use of electronic patient portals, but also to standardize and expand how patients are offered access so that diverse and vulnerable patient populations are consistently invited to access their records online.

Within each focus group one or two individuals strongly advocated the benefits of using portals and offered to help interested non‐users. The opportunity to connect patients with limited computer literacy with tech‐savvy peers suggests a potential method for addressing this barrier to obtaining electronic health information among vulnerable populations. Peer navigators have been effective advocates for patients who have challenges using complex health systems,^24^ and have demonstrated benefit for patients with diabetes and multiple chronic conditions.^25^, ^26^, ^27^ Peer navigators with diabetes who are also facile with portal use could assist interested patients in obtaining their own electronic health information and help identify and relate the information that is important for diabetes self‐care. This novel additive component of peer navigation in health systems with electronic portals for safety‐net populations should be studied with the goal of narrowing the “digital divide.”

Health literacy is identified by the Agency for Health Research and Quality (AHRQ) as an important patient safety and quality measure, and opening access to electronic written notes provides an opportunity to improve health literacy. Low literacy patients could review the notes with their provider; they could share them with a caregiver or family member; and they could read them after the visit slowly in a low‐pressure environment. Our safety‐net focus group participants reported that the notes and After‐Visit Summaries were clear and helpful, while simultaneously identifying aspects that were inaccurate or confusing; many particularly reported wanting to have all the details in the notes. Positive themes about access to electronic notes were also reported in a focus group study in a Veterans Administration population, including the desire not to sacrifice detail for simplification.^28^ In a qualitative study in the UK patients' interactions with health‐care providers were thought to be improved by access to health records,^29^ which our participants also noted. Patients relied on their providers to interpret unclear concepts or medical jargon. As electronic health records evolve, options for including definitions for commonly used medical terminology and abbreviations will be useful for some patients; and some patients will always rely on verbal communication to clarify the plan, but can use the written notes or summaries as a vehicle to share the information with others who are involved in their care. Augmenting health literacy by building patient/provider trust and communication with transparent notes will likely benefit all populations, including those in the safety‐net and with low literacy.

Patients identified inaccuracies in their doctors' notes and After‐Visit Summaries, a finding consistent with prior OpenNotes research,^30^ which demonstrates a distinct advantage of allowing patients to review their records. As patients' access to their online health record increases nationally, gathering their feedback on the content of clinic notes has the potential to improve the accuracy of clinical documentation, promote patient engagement, and improve overall patient outcomes.^31^, ^32^, ^33^ One opportunity for decreasing errors includes patients directly entering or editing content into their own electronic record. A recent study in which patients at a safety‐net clinic were asked to type their agenda for their clinic visit into an electronic health record demonstrated that patients were interested and able to do so.^34^ Re‐design of the way that doctors' notes and After‐Visit Summaries are constructed by the electronic health record is another potential avenue for decreasing inaccurate information. Auto‐generation of notes and After‐Visit Summaries with templates and pre‐populated data are a common feature of electronic health records, including the one used at our study sites. Focus group participants noted inaccuracies primarily in the problem list and medication sections of their documents; fields that are typically “pulled in” from chart sections that are cumbersome for doctors and staff members to modify. Our findings support the need to re‐engineer the clinic note and physician workflows in electronic records, so that the data are easily modified for accuracy.^35^ Electronic health records should support efficient physician workflows for maintaining correct health data, and partner with patients to ensure accuracy, which subsequently would improve care delivery and prevent avoidable errors.

Doctors have historically written notes for communication with medical colleagues, while After‐Visit Summaries have been developed explicitly for patients to read. As more patients gain access to their doctors' notes, there is opportunity to consider adjustments to note content and clarity so that they are more useful to patients,^36^ while maintaining the clinical detail necessary for accurate capture of the encounter. For many patients, the After‐Visit Summary information may suffice, but the option to read the full content of the note may encourage trust and transparency, and provide detail needed for complex or multiple chronic conditions.

This study was limited by the small sample size necessitated by the qualitative focus group format. Most patients were recruited from pre‐existing clinic registries and were chosen by clinic staff members for their potential to participate, which may attract those who are more engaged in their care and thus see more potential benefit from accessing their health information online. Patients all carried a diagnosis of diabetes mellitus and one focus group was composed of patients with diabetes and HIV; thus, study results may not be generalizable to populations without a chronic disease that requires regular contact with providers and the medical system.

Despite these limitations, this study provides meaningful insights to help improve access to electronic health information among vulnerable populations, including patient‐identified barriers to online access and patient portal use. One strength of the study design—having patients read their recent clinical documents immediately before the group discussions—was that it elicited more detailed and targeted feedback about their own doctors' notes. The majority of participants reported they found the note content clear and useful on the survey. However, during the focus group discussions, participants acknowledged that there were aspects of the note that they did not understand, such as medical jargon and abbreviations, which might not have been disclosed otherwise. Additional research is needed into potential solutions, such as facilitating online access, training patients about key features of the portal, explaining privacy features and utilizing peer educators or navigators. There are ample opportunities to engage patients to help ensure medical records are clear, accurate and useful to them, and their family members, caregivers and their other providers.^37^ Vulnerable patient populations should be explicitly included in efforts to integrate patient feedback.

Developments in health information technology have dramatically changed the way health care is delivered, but there are still hurdles which prevent patients from fully benefitting from online health records. This is particularly true for vulnerable patients, many of whom are unable to access online patient portals, while others are resistant due to privacy concerns or a lack of interest or understanding in the content of their online records. Electronic patient portals create both challenges and opportunities for patients to participate in their care, and all stakeholders should be thoughtful in developing and implementing this technology so as not to aggravate existing health disparities.

## CONFLICT OF INTERESTS

No conflict of interests have been declared.
