# Supplementary material for: Patients' perceptions of their doctors' notes and after‐visit summaries: A mixed methods study of patients at safety‐net clinics
Source: Health Expect. 2017 Nov 2;21(2):485–93. doi: 10.1111/hex.12641 (PMC5867322; doi:10.1111/hex.12641)
Supplement: Supplementary file 1 [file HEX-21-485-s001.docx]

**Supplementary Appendix**

**Appendix 1**. Focus Group Survey

First Name______________ Clinic___________________ Date:_________________

**Focus Group Survey**

| **1. Please answer the following questions about your *After Visit Summary*:** | | | | | |  |
| --- | --- | --- | --- | --- | --- | --- |
|  | Strongly Disagree | Disagree | Neutral | Agree | Strongly Agree | Not Sure |
| 1. After reading my *After Visit Summary*, I **understand my plan of care.** |  |  |  |  |  |  |
| 1. My *After Visit Summary* contains **content that is useful.** |  |  |  |  |  |  |
| 1. My *After Visit Summary* has a **clear treatment plan.** |  |  |  |  |  |  |
| 1. My *After Visit Summary* has a **clear medication list.** |  |  |  |  |  |  |
| 1. My *After Visit Summary* is **too long**. |  |  |  |  |  |  |
| 1. My *After Visit Summary* has **too much medical jargon/too much doctor language.** |  |  |  |  |  |  |
| 1. My  *After Visit Summary* contains information that is **NOT accurate.** |  |  |  |  |  |  |
| 1. If you have other comments about your  *After Visit Summary*, write them here. |  | | | | | |

| **2. Please answer the following questions about your *Clinic Note*:** | | | | | |  |
| --- | --- | --- | --- | --- | --- | --- |
|  | Strongly Disagree | Disagree | Neutral | Agree | Strongly Agree | Not Sure |
| 1. After reading my *Clinic Note*, I **understand my plan of care.** |  |  |  |  |  |  |
| 1. My *Clinic Note* contains **content that is useful.** |  |  |  |  |  |  |
| 1. My *Clinic Note* has a **clear treatment plan.** |  |  |  |  |  |  |
| 1. My *Clinic Note* has a **clear medication list.** |  |  |  |  |  |  |
| 1. My *Clinic Note* is **too long**. |  |  |  |  |  |  |
| 1. My *Clinic Note* has **too much medical jargon/too much doctor language.** |  |  |  |  |  |  |
| 1. My *Clinic Note* contains information that is **NOT accurate.** |  |  |  |  |  |  |
| 1. If you have other comments about your  *Clinic Note*, write them here. |  | | | | | |

**3.) I have accessed my medical record through eCare:**

Yes

No

**a.) If No, why not (check all that apply):**

No computer or other electronic device to access it

Have computer/other device, but too complicated to access

Not interested

Concern that the information would make me worry

Concern about my privacy

Other __________________________

**b.) If Yes, what did you read or use on eCare (check all that apply):**

- Visit notes
- Labs
- Radiology
- Sent message to my provider
- Pharmacy refills
- Other __________________________

The following questions are to help us understand the demographics of the group attendees. You can choose to skip any questions that you do not feel comfortable answering.

**4.) What is your gender?**

- Female
- Male

**5.) How old are you? ______**

**6.) What is your race (please select one that you feel best applies)?**

American Indian or Alaska Native

Asian

Black or African American

Native Hawaiian or other Pacific Islander

White

Other (Specify: ______________________)

**7.) Are you Hispanic/Latino(a)?**

Yes

No

**8.) How many people do you live with? (Insert Number – Enter “0” if you live alone)** ______

**9.) What is your relationship status?**

Single  Not Married-living together

Married  Widowed

Divorced  Separated

**10.) What is your current employment status?**

Employed Full-time

Retired

Unemployed

Employed Part-time

Disabled

Other (please describe) __________

**11.) What is the highest grade you completed?**

8^th^ grade or less

Some high school  High school graduate or equivalent (GED)

Some college  College graduate

Graduate or professional school

**12.) In general how would you rate your overall health (choose only ONE)?**

- Excellent
- Very Good
- Good
- Fair
- Poor

**13.) Check off all of the body systems that your diabetes has affected?**

- Eyes (for example, retinopathy)
- Kidneys (for example, nephropathy)
- Skin (for example, foot ulcers)
- Heart/Blood vessels (for example, heart attack or stroke)
- Feet/Legs (for example, amputations)

| **14. I am confident in my ability to manage by diabetes.** | | | | | |
| --- | --- | --- | --- | --- | --- |
| Strongly Disagree | Disagree | Neutral | Agree | Strongly Agree | Not Sure |
|  |  |  |  |  |  |

Thank you for completing this survey.
